# Dysregulation in the Unfolded Protein Response in the FGR Rat Pancreas

**DOI:** 10.1155/2020/5759182

**Published:** 2020-01-20

**Authors:** Xiaomei Liu, Yanyan Guo, Jun Wang, Liangliang Zhu, Linlin Gao

**Affiliations:** ^1^Key Laboratory of Maternal-Fetal Medicine of Liaoning Province, Department of Obstetrics and Gynecology, Shengjing Hospital, China Medical University, Shenyang 110004, China; ^2^Department of Obstetrics and Gynecology, Benxi Central Hospital of China Medical University, Benxi 117022, China

## Abstract

Accumulating evidence suggests that fetal growth restriction (FGR) leads to the development of diabetes mellitus in adults. The aim of this study was to investigate the effect of protein malnutrition *in utero* on the pancreatic unfolded protein response (UPR) pathway in FGR offspring. An FGR model was developed by feeding a low-protein diet to pregnant rats throughout gestation. Eighty-four UPR pathway components in the pancreas were investigated by quantitative PCR arrays and confirmed by qPCR and western blotting. Activating transcription factor (Atf4 and Atf6), herpud1, protein kinase R-like endoplasmic reticulum kinase (Perk), X-box binding protein 1 (Xbp1), and the phosphorylation of eIF2*α* were upregulated, while cyclic AMP-responsive element-binding protein 3-like protein was markedly downregulated in FGR fetuses compared with controls. Investigation in adult offspring revealed temporal changes, for most UPR factors restored to normal, except that dysregulation of Atf6 and Creb3l3 maintained until adulthood. Moreover, autophagy was suppressed in FGR fetal pancreas and may be associated with decreased activation of AMP-activated protein kinase (Ampk). Apoptosis regulators Bax and cleaved-caspase 3 and 9 were upregulated in FGR fetal pancreas. Given that islet size and number were decreased in FGR fetus, we speculated that the aberrant intrauterine milieu impaired UPR signaling in fetal pancreas development. Whether these alterations early in life contribute to the predisposition of FGR fetuses to adult metabolic disorders invites further exploration.

## 1. Introduction

Fetal growth restriction (FGR) is the second leading cause of perinatal death after prematurity and affects 3–7% of all pregnancies [1, 2]. FGR is largely attributed to an insufficient oxygen and nutrient supply by the placenta, such that embryonic tissues and organs fail to grow to their normal size [3]. It is becoming apparent that FGR has major impacts on the fetus, with negative effects on cardiovascular, metabolic, and neurological development. FGR increases the susceptibility to metabolic syndrome (type 2 diabetes mellitus (T2DM), obesity, and cardiovascular diseases) [4–6]. FGR fetuses have reduced circulating insulin levels, hyperglycemia, and impaired glucose tolerance [7]. Using a well-established rat models caused by low-protein (LP) diet, our previous work has suggested that maternal malnutrition leads to hyperglycemia and impaired glucose tolerance in FGR adult rats [8], consistent with other reports [9, 10].

It is well established that FGR is an independent risk factor for the development of T2DM, although the mechanisms underlying the impacts on pancreatic development and insulin sensitivity are unclear, with conflicting results among different animal models. In most reports, FGR fetuses caused by a low-calorie or LP diet during pregnancy are characterized by a reduced level of glucose-stimulated insulin secretion, reduced pancreas size, small islets with fewer *β*-cells, and impaired pancreatic vascularity [11]. Moreover, *α* cell masses are reduced in FGR fetuses [12]. However, enhanced glucose-stimulated insulin secretion and insulin sensitivity are also present in young lambs with hyperthermia-induced FGR [13].

External factors, such as malnutrition and lipid overload, lead to protein folding disorders. Unfolded/misfolded proteins accumulate in the endoplasmic reticulum (ER), disrupt ER homeostasis, and trigger a protective response termed the unfolded protein response (UPR) [14, 15]. The UPR involves three chief pathways that are regulated by the following transmembrane ER proteins: PKR-like ER kinase (PERK), activating transcription factor (ATF) 6, and inositol-requiring protein (IRE) 1 [16–18]. The UPR is primarily a protective response, triggering the production of ER molecular chaperones and stress response proteins to enhance protein degradation and increase the protein folding capacity of the ER [14]. However, an unresolved UPR can trigger apoptotic or autophagic machinery and ultimately lead to cell death [19].

ER stress is closely linked to a range of metabolic disorders, including diabetes and obesity, and causes pancreatic *β*-cell dysfunction and apoptosis as well as peripheral tissue insulin resistance [20]. The UPR is also associated with maternal programming. Maternal obesity triggers alterations in the UPR signaling pathway in hepatic and pancreatic tissues [21, 22]. Uterine artery ligation upregulates Atf6 and p-eIF2 in the adipose tissues of juvenile FGR rats, which contributes to the development of glucose intolerance [23]. We previously showed that a maternal LP diet triggers alterations in the UPR pathway in the liver of FGR progeny [24]. Using the same model, we evaluated the impact of FGR on the UPR pathway in the pancreas with the aim of elucidating the mechanisms underlying developmental abnormalities of the pancreas and hyperglycemia.

## 2. Materials and Methods

### 2.1. Animals

Female Wistar rats were randomly divided into two groups after mating with male rats. The animals in the undernourished group were fed an isocaloric low-protein diet (7% protein, according to the standard of AIN-93 purified diet for laboratory rodents from American Institute of Nutrition) throughout the pregnancy period, as previously described [24], while the control animals were maintained on a standard chow diet (22% protein) (Supplement Table 1). At 20 days of gestation (E20, near term), a set of pups were delivered by caesarian section. Here, FGR refers to fetus with a birth weight of at least two standard deviations lower than the average birth weight of normal fetus, and those birth weight did not meet the criteria were excluded. Other pregnant rats were allowed to deliver spontaneously and only litters with 10–14 pups were selected for subsequent experiments, to exclude the effects of unbalanced development caused by litter size. The fetal pancreas from each litter was obtained, pooled (by combining three samples), and stored until analysis. Juvenal rats were weaned at 3 weeks of age, and then all fed with standard rat chow until 12 weeks (12 W). Only male adult offsprings were used to avoid interference from sex-related differences. At 12 W, offspring rats were killed under ether anesthesia, and the pancreas was collected and stored at 80°C until analysis. Animals were maintained under specific pathogen-free conditions with a 12 h light/12 h dark cycle at 22°C with food and water provided ad libitum throughout the study period.

### 2.2. Histological Staining

Fetal pancreases were formalin-fixed and paraffin-embedded before sectioning. The 3 *µ*m sections were subjected to immunohistochemical (IHC) staining according to standard procedures. In brief, after antigen retrieval and blocking for endogenous peroxidase and nonspecific binding, the sections were incubated with primary antibodies (Supplement Table 2) overnight at 4°C and blotted with the biotinylated secondary antibody (Beyotime, Haimen, China). Pancreas sections were also subjected to immunofluorescence (IF) according to standard procedures with Perk antibody. A negative control without the primary antibody was also included, and no background staining was observed. Imaging was performed using a Nikon ECLIPSE Ti microscope (Tokyo, Japan). For each group, up to five sections (each containing at least five islets) of each pancreas were chosen for the evaluation of immune-positive areas. In each group, pancreases of up to 3 rats were examined.

### 2.3. Quantitative PCR (qPCR) Array

The mRNA levels of UPR-related molecules (including 84 UPR-related genes, 5 housekeeping genes, and 7 quality control genes; Supplementary Table 4) in the fetal pancreas were measured using qPCR arrays (PARN-089Z; QIAGEN, Frederick, MD, USA) as previously described [24]. To reduce intersample variation, nine controls from three litters were pooled into three samples (control 1, control 2, and control 3), and nine FGR samples from three litters were pooled to obtain FGR1, FGR2, and FGR3. In brief, total RNA was extracted from the fetal pancreas, quantified, and used for integrity detection. The cDNA was synthesized, and PCR assays were performed in 96-well PCR plates. The global patterns of UPR-related molecules were analyzed by Student's *t*-tests and visualized using *K*-means clustering in conjunction with a heatmap and volcano plot.

Routine qPCR techniques were used to validate the PCR array results with a larger sample size (*n* = 6–8). In brief, total RNA was extracted, quantified, and reverse transcribed. Quantitative real-time PCR was performed using the ABI PRISM 7500 HT Fast Real-time PCR System (Applied Biosystems, Austin, TX, USA) and SYBR Green PCR Kit (Novizan, Q711-02, Nanjing, China) with gene-specific primers, which was listed in Supplement Table 3. The analysis of mRNA level was performed using the 2^−ΔΔ*Ct*^ method after normalization, with *β*-actin as an internal control.

### 2.4. Western Blot Analysis

Total proteins were extracted from the pancreas with SDT buffer (4% sodium dodecyl sulfate (SDS), 1 mM dithiothreitol (DTT), 150 mM Tris-HCl, pH 8.0). UPR-related protein expression was determined by western blot analyses. Total protein was loaded onto a 10–12% polyacrylamide gel for electrophoresis and transferred to a polyvinylidene membrane by electroblotting. After blocking with 5% BSA in TBS-T, the membrane was divided into several strips to detect different target proteins depending on the molecular weight (Supplement Table 2). The blots were then incubated with appropriate secondary antibodies and developed using Immobilon Chemiluminescent HRP substrate (EMD Millipore Corporation, Burlington, MA, USA) on a ChemiDoc XRS Imaging System (Bio-Rad Laboratories, Hercules, CA, USA). The optical density of bands was quantified using Gel-analyzer software.

### 2.5. Statistical Analysis

Data are presented as means ± standard error of the mean (SEM). All data were checked for normal distribution using the Shapiro–Wilk test. The statistical analyses were performed by a two-tailed, unpaired Student's *t*-test implemented in SPSS 17.0 (SPSS Inc., Chicago, IL, USA) for parametric data. Statistical significance was accepted at *p* ≤ 0.05.

## 3. Results

### 3.1. Effect of Protein Malnutrition on Pancreas Histopathology

**Figure 1:**
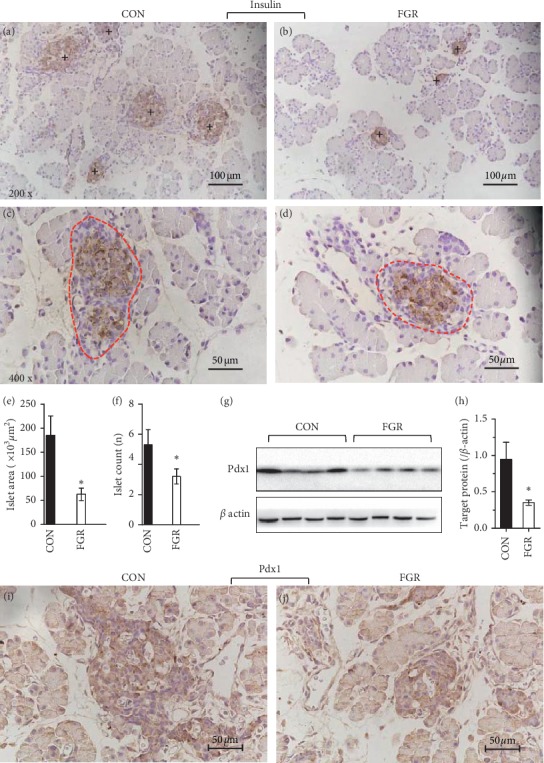
Fetal pancreatic histopathology. (a–f) Analysis of the islet index by insulin staining. Representative photomicrographs of IHC staining for insulin at 200x (a, b) or 400x (c, d) magnification in the pancreas of control (a, c) and FGR (b, d) fetuses. (e–f) Analysis of the islet index. Islet area (e) were calculated based on the photomicrographs at a magnification of 400x. (f) Islet count was calculated based on the photomicrographs (black cross marks the islet) at 200x. (g, h) Representative photomicrographs of immunoblotting results for Pdx1, an *\* cell marker. Data are expressed as means ± SEM. ^*∗*^*p* < 0.05 vs. controls (*n* = 5-6). (i, j) Representative photomicrographs of IHC staining for Pdx1 at 400x magnification in the pancreas of control (i) and FGR (j) fetuses.

In both groups, fetal islets were structurally intact with clear boundaries and scattered in the exocrine acinus of the pancreas showed by IHC with insulin antibody. Most of the islet cells were insulin-positive (Figures 1(a)–1(d)). Compared with control, the FGR fetus was characterized by remarkable decreases in the pancreatic islet counts and mass size (Figures 1(e) and 1(f)). The protein level of Pdx1, a *β*-cell marker which is essential for pancreatic development [25], was significantly reduced in the FGR pancreas (Figures 1(g)–1(j)).

### 3.2. Effect of Protein Malnutrition on Fetal Pancreatic mRNA Levels of UPR Pathway

**Figure 2:**
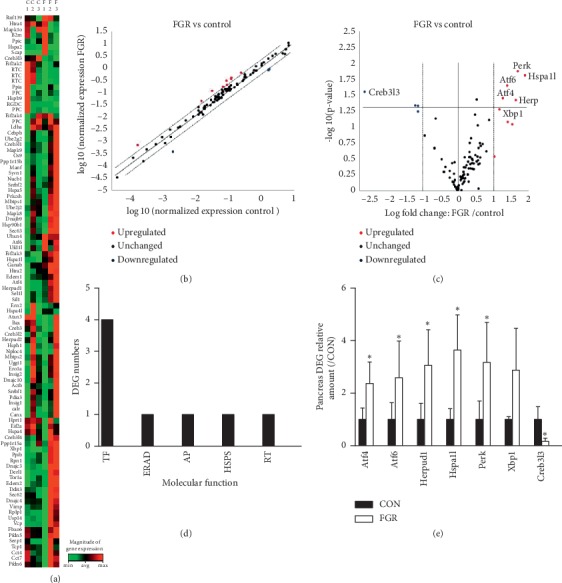
Expression of unfolded protein response- (UPR-) related factors in the FGR fetal pancreas, as determined by qPCR arrays. (a) *K*-means clustering representation of eighty-four mRNA profiles. The magnitude of the change is represented by a color scale (top right) from low (green) to high (red). b and c indicates scatter plot and volcano plot displaying differentially expressed genes in pancreatic tissues between two groups (*n* = 3). (b) The *x* and *y* axis, respectively, indicate the mean expression value of control and FGR after log_10_ transformation. (c) The *y*-axis indicates the *p* value after log_10_ transformation, and the *x*-axis displays the log2 fold change (FC) value (FGR/control). Red dots represent upregulated transcripts (FC > 2); blue dots represent downregulated transcripts; black dots represent unchanged transcripts (0.5 < FC < 2). (d) Differentially expressed genes divided into 5 categories according to molecular function. TF, transcription factor; ERAD, endoplasmic reticulum-associated degradation; AP, apoptosis, HSPs, heat-shock protein; RT, regulation of translation. (e) Differentially expressed genes between two groups (*n* = 3 per group; ^*∗*^*p* < 0.05 vs. control, unpaired Student's *t*-test).

**Figure 3:**
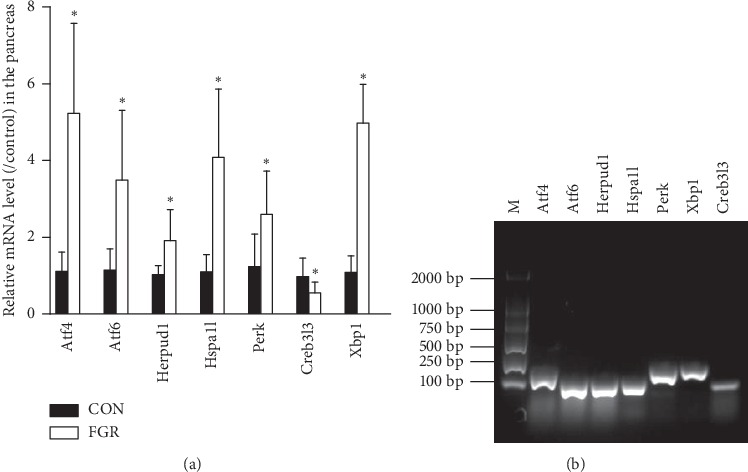
Determination of differentially expressed genes in UPR pathways in the fetal pancreas. (a) qPCR analysis showing the mRNA levels of pancreatic UPR-related genes in fetal rats. Data are expressed as means ± SEM. ^*∗*^*p* < 0.05 vs. controls (*n* = 6–8). (b) Representative agarose gel electrophoresis images of PCR products confirming amplicon specificity.

We analyzed qPCR array data using a fold change cutoff of >2 and a significance threshold of *p* ≤ 0.05. The significance and magnitude of differences between groups were evaluated by *K*-means clustering and visualized using heatmaps (Figure 2(a)), scatter plots (Figure 2(b)), and volcano plots (Figure 2(c)). Seven of the eighty-four UPR molecules displayed significant differential transcription level; 6 were upregulated; and 1 was down-regulated in the FGR fetal pancreas compared with the control. These differentially expressed molecules could be assigned to five categories of UPR pathway according to molecular function, including transcription factor, endoplasmic reticulum-associated degradation, and regulation of translation, apoptosis and heat-shock protein (Figure 2(d)). Specifically, the mRNA levels of Atf2, Atf6, Herp, Hspa1l, and Perk were significantly higher in the FGR fetal pancreases than in the controls, while Creb3l3 level was remarkably lower (*p* < 0.05) (Figure 2(e)). Pancreatic X-box binding protein 1 (Xbp1) tended to be highly expressed in the FGR fetuses, but the difference was not significant (*p*=0.092), owing to high intersample variation. With an increased sample size, qPCR confirmed the results of the PCR array. Overall, protein restriction *in utero* significantly increased pancreatic Atf2, Atf6, Herp, Hspa1l, Xbp-1, and Perk mRNA levels and decreased Creb3l3 transcription (Figure 3(a)). PCR product specificity was confirmed by electrophoresis on 2% agarose gels stained with Goldview (Figure 3(b)).

### 3.3. Effect of Protein Malnutrition on Fetal Pancreatic Protein Levels of UPR Factors

**Figure 4:**
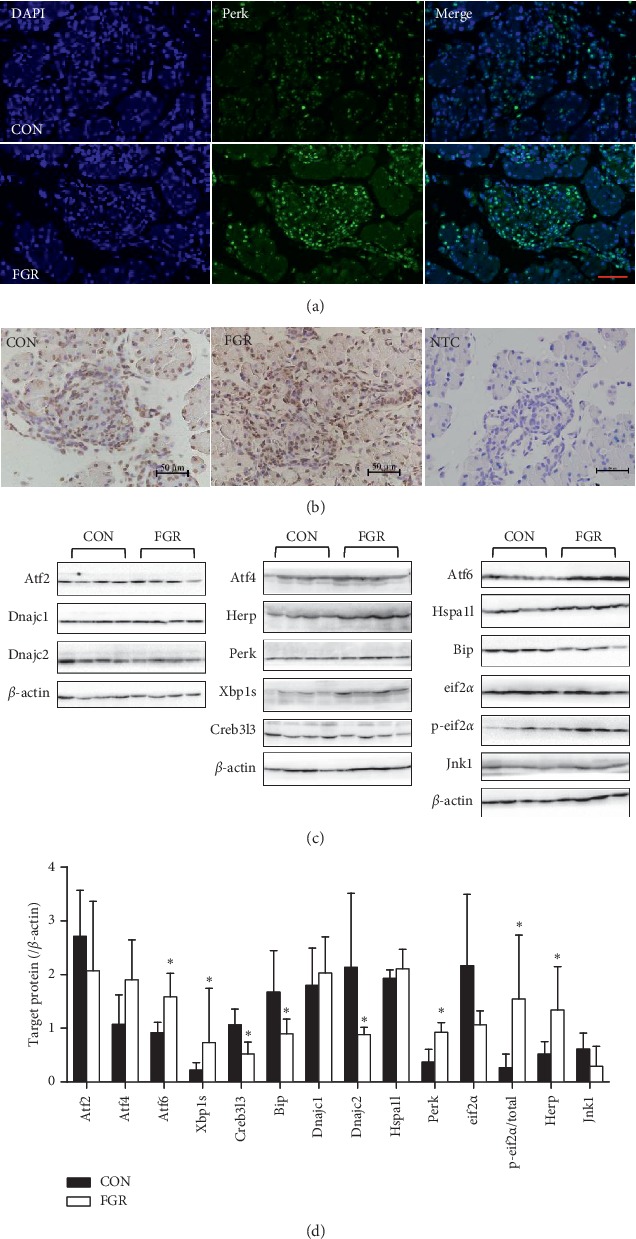
Protein levels of pancreas UPR molecules determined by western blotting. Representative photomicrographs of (a) immunofluorescence analysis of Perk in sections of the fetal pancreas from control (upper panel) and FGR (lower panel) (original magnification 400x); (b) IHC staining of Perk in fetal pancreas from both groups; NTC, negative control (magnification: 400x; scale bar = 50 *μ*m); and (c) immunoblotting for UPR-related factors at E20. (d) Statistical analysis of the protein levels at E20. Data are expressed as means ± SEM. ^*∗*^*p* < 0.05 vs. controls (*n* = 6–8, unpaired Student's *t*-test). UPR molecules divided into 5 categories according to molecular function. TF, transcription factor; ERAD: endoplasmic reticulum-associated degradation; AP, apoptosis; HSPs, heat-shock protein; RT, regulation of translation.

The impact of maternal protein malnutrition was further evaluated on the protein levels of UPR molecules. Transcription factor Perk mainly distributed in the nucleus and seemed more highly expressed in FGR fetuses evidenced by IHC and IF (Figures 4(a) and 4(b)). Immunoblotting confirmed that Perk protein was significantly upregulated in FGR fetuses than in controls. eIF2*α*, a key Perk mediator, showed no significant difference in total protein levels, while the phosphorylation of eIF2*α* was significantly elevated in the FGR group, resulting in a significant increase in the ratio of p-eIF2*α* to total eIF2*α* (Figures 4(c) and 4(d)). Atf4, a target of the Perk-eIF2*α* pathway, tended to be higher in the FGR pancreas than in the control, but the difference was not statistically significant (*p*=0.053). Atf4 regulates CHOP expression, inducing the activation of JNK and leading to ER stress and apoptosis. Modest upregulation of Atf4 may not be enough to affect downstream molecules; neither Chop mRNA (Supplemental Table 4) nor Jnk1 protein showed significant differences between groups. Interestingly, there was no difference in the hspa1l protein level between the two groups at E20 (Figures 4(c) and 4(d)), although the mRNA levels were elevated in the FGR group.

Atf6, another UPR upstream transducer, exhibited significantly higher protein levels in the FGR fetal pancreas, while Creb3l3 protein level was remarkably lower. There was no statistical difference in the Ire1*α* mRNA level (Supplemental Table 4). However, maternal low-protein level led to an increase in the levels of Xbp-1 mRNA (Figure 3(a)) and spliced Xbp1 protein. Consequently, Herp, which is induced by IRE1*α*-Xbp1 and Atf6, showed an increase in the FGR group. Both Dnajc2, a cytosol chaperone, and BIP/Grp78, an ER chaperone protein were decreased in FGR fetus (Figures 4(c) and 4(d)), despite similar mRNA levels in the two groups. It suggests that malnutrition *in utero* affects some UPR factors at the translational or posttranslational levels, rather than at the transcriptional level.

### 3.4. Expression of Autophagy and Apoptosis Regulators in Fetal Pancreas

**Figure 5:**
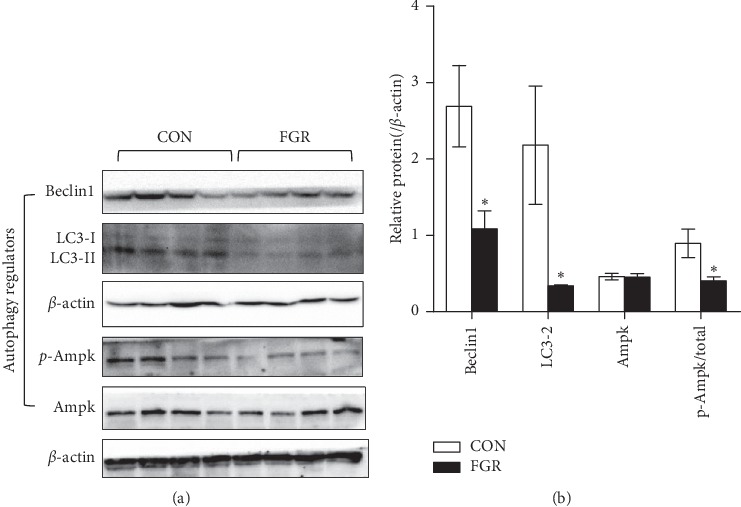
Determination of autophagy regulators in fetal pancreas. (a, b) Representative immunoblotting and densitometric quantification of autophagy regulators in the fetal pancreas. Data are expressed as means ± SEM. ^*∗*^*p* < 0.05 vs. controls (*n* = 6–8, unpaired Student's *t*-test).

**Figure 6:**
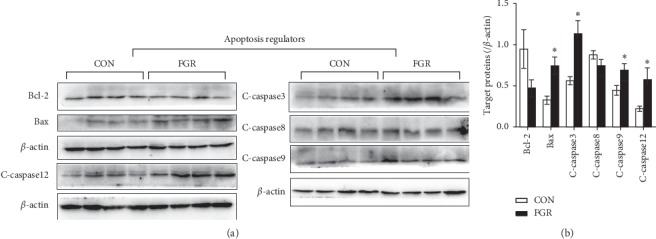
Determination of apoptosis regulators in fetal pancreas. (a, b) Representative photomicrographs and statistical analysis of apoptosis regulators in the fetal pancreas Data are expressed as means ± SEM. ^*∗*^*p* ≤ 0.05 vs. controls (*n* = 4–6, unpaired Student's *t*-test). C-caspase: cleaved-caspase.

Autophagy regulators Beclin 1 and LC3-II were remarkably decreased in the fetal pancreas. AMP-activated protein kinase (AMPK), an autophagy promoter showed lower phosphorylation level in the FGR group (Figures 5(a) and 5(b)). Immunoblotting showed that proapoptotic protein Bax was remarkably higher in FGR group, compared with the control group, while antiapoptosis protein Bcl-2 was unchanged (Figures 6(a) and 6(b)). We also observed significant higher levels of cleaved caspases 3, 9, and 12 in the FGR group, compared with control fetus (Figures 6(a) and 6(b)).

### 3.5. Expression of UPR Genes in Adult Pancreas

**Figure 7:**
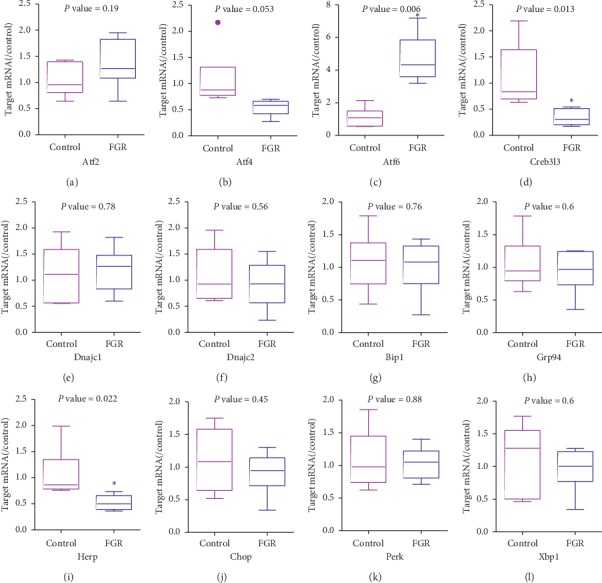
Determination of UPR genes in adult offspring. Q-PCR analysis of key UPR factors in the adult pancreas. Data were expressed as the mean ± SEM. *n* = 6, ^*∗*^*p* < 0.05 vs. age-matched control.

To explore whether the changes in pancreas were transient or permanent, the UPR pathway was further determined in adult (12W) offspring. Q-PCR assay showed that mRNA level of most UPR genes including Atf2, Dnajc1/2, Grp78/94, Chop, Perk, and Xbp1 was similar with age-matched control at 12 W, which indicated a temporal window of vulnerability. However, increase in Atf6 and decrease in Creb3l3 were maintained until 12 W. Contrary to the trend of the fetus, the mRNA levels of Herp were significantly decreased in adult FGR pancreas (Figure 7).

## 4. Discussion

Epidemiological and experimental studies have substantiated the proposed link between adverse intrauterine environments and adult diseases, such as T2DM and hyperglycemia. This study demonstrated that FGR fetus caused by a low-protein diet during pregnancy was characterized by reduced pancreatic islet numbers and size. Alterations in FGR fetus might be due to protein deficiency, though we could not rule out the possibility that slightly higher carbohydrates and fat in the isocaloric low protein diet may also have an independent impact on pancreas development.

Previous report suggested that FGR induced by a uteroplacental insufficiency triggers the activation of hepatic UPR in offspring rats, indicating that UPR signaling play a role in the fetal programming of metabolic diseases [26]. This study demonstrated that major UPR pathways in pancreas were dysregulated by protein deficiency *in utero*, but most UPR factors restore to normal in adulthood. Using the same model, we previously reported that blood glucose and plasma insulin level were lower in FGR fetus [27], but higher in adulthood [8]. We speculate that lower insulin levels in FGR fetus was due to fetal pancreatic dysplasia. Increased insulin levels in adulthood may be due to significant insulin resistance in tissues such as the liver and skeletal muscle, which has been confirmed by several reports [28, 29].

In the FGR pancreas, Perk, a UPR transducer, was upregulated by ER stress and subsequently phosphorylated eIF2, which represses the translation of most mRNAs in the ER but selectively increases the translation of ATF4, resulting in the induction of the downstream gene Chop [30]. Atf4 mRNA levels were significantly higher in the FGR pancreas, consistent with expectations. However, there was no significant difference in Atf4 protein levels between two groups. Accordingly, Chop was unchanged at the mRNA level. Atf4 gene expression levels were not correlated with protein levels, suggesting posttranscriptional regulation or possible rhythmical activation of the UPR pathway [22], which deserves further research. Molecular chaperone was involved in folding or maintaining of nascent polypeptides [31]. We speculated that insufficient chaperone Dnajc2 and Bip lead to incorrect folding or aggregation of polypeptides and then disrupt ER homeostasis in FGR fetus [32].

ATF6 plays a major role in the regulation of ER quality control proteins by regulating GRP78 and XBP1 [33]. Our results indicated that FGR lead to a significant increase in ATF6 activation, thereby increasing ER stress in the FGR pancreas. Atf6 augments the expression of Xbp1 mRNA, providing more substrates for the IRE1-induced generation of Xbp1s [34]. Further research showed that increased Xbp1 mRNA transcription is induced by Atf6 in the FGR pancreas, though there were no changes in Ire1 mRNA levels. In the FGR pancreas, upregulated Atf6 and Xbp1 also induce an increase in Herp, a ubiquitin-like membrane protein that is abundant in the pancreas [35], suggesting an important role in the degradation of ER proteins in the pancreas.

A maternal low-protein diet reduced the expression of Creb3l3 in the fetal pancreas and maintained until adulthood. Interestingly, we previously showed upregulated Creb3l3 in hepatocytes of the FGR fetus using the same model. Creb3l3 is considered as an ER stress-associated liver-specific transcription factor, and most studies focused on its function in the hepatocytes. In response to ER stress, Creb3l3 is cleaved by S1P and S2 and translocate into nucleus [36] and transactivates acute phase response (APR) pathway [37] and hepcidin expression in hepatocytes [38, 39]. Creb3l3 elicits UPR signaling in a tissue-specific manner. It is expressed at lower levels in human islets. It mediates the adaptive UPR in response to FFAs, but is reduced in cases of severe chemical ER stress [40]. Opposite changes in Creb3l3 in different tissues suggest a novel role for Creb3l3 in pancreas UPR signaling.

It is well established that UPR activation lead to changes of key autophagy regulators. PERK/eIF2*α* pathway induces the expression of autophagy-related genes through ATF4, and XBP1 activation leads to the conversion of soluble LC3-I to lipid bound LC3-II and the formation of autophagosomes [41]. Nevertheless, autophagy marker LC3-II was reduced in FGR pancreas, contrary to our expectation. Beclin 1, a core component playing a central role in the regulation of autophagy was also decreased. AMPK signaling is a major positive regulatory axis of autophagy and direct inhibition of AMPK activity is sufficient to suppress autophagy induction. We speculated that the suppression of autophagy resulted from lower phosphorylation of AMPK in the pancreas of FGR fetus, which is yet to be proved by further research.

Prolonged ER stress impairs insulin synthesis and causes pancreatic *β*-cell apoptosis. Pdx1 deficiency enhances *β*-cell susceptibility to ER stress-associated apoptosis [42], Our work revealed decreased PDX protein in the IUGR fetal pancreas. Further research showed an increase in proapoptosis protein Bax and in activated caspase 3, 9, and 12 in IUGR fetal pancreas, consistent with our speculation.

One limitation of our research is using the whole pancreas for analysis. The pancreas is composed of both exocrine and endocrine tissues (islet). Human islets consist of ∼60% *β* cells and 30% *α* cells, the remaining 10% consisting of δ cells, *γ* cells and ϵ cells. Malenvironment *in utero* have different effects on endocrine and exocrine cells, so isolating different cells of the pancreas for study will be more precise. IUGR induced by uterine artery ligation also led to temporal changes in IUGR islets. Both extracellular matrix genes and mesenchymal stromal cell-derived factors were increased at 2 weeks and decreased in adulthood [43]. We speculated that the discrepancy between two results derived from different rat models (uteroplacental insufficiency *vs.* LP) and different developmental stages (2, 10 weeks *vs.* embryo 20 days)and different tissues (islet *vs.* whole pancreas). These results indicated that IUGR caused by different aetiologies lead to different changes in different cells and also one type of rat model cannot completely simulate the condition of human diseases.

In summary, our findings demonstrated that maternal protein malnutrition triggers an ER stress imbalance and leads to disruptions in the UPR pathway, which might be responsible for fetal pancreatic dysplasia. Further studies of nutritional epigenetic programming of ER stress are warranted, given the potential implications for early development and treatment strategies to reduce the incidence of metabolic syndrome in adults.

## Data Availability

The data used to support the findings of this study are available from the corresponding author upon request.

## Abbreviations

FGR: Fetal growth restriction
HSPA1L: Heat-shock 70-kDa protein 1 L
CREB3L: Cyclic AMP-responsive element-binding protein 3-like protein
EIF2: Eukaryotic translation initiation factor 2
UPR: Unfolded protein response
GRP: Glucose-regulated protein
IRE1: Inositol-requiring enzyme 1
ATF6: Activating transcription factor 6
PERK: Protein kinase *R*-like endoplasmic reticulum kinase
HERPUD1: Homocysteine-responsive endoplasmic reticulum-resident ubiquitin-like domain member 1 protein
CHOP: C/EBP homologous protein
ERAD: ER-associated protein degradation
XBP1: X-box binding protein 1
PDX1: Pancreas/duodenum homeobox protein 1
LC3: Microtubule-associated protein 1 light chain 3
AMPK: AMP-activated protein kinase.

## Supplementary Materials

Supplementary Materials Supplement Table 1: composition of rat diets. Supplement Table 2: antibodies for western blotting, IHC, and IF. Supplement Table 3: primers for qPCR. Supplement Table 4: relative expression of the 84 UPR-related genes by PCR Arrays.

## References

[1] Romo A., Carceller R., Tobajas J. (2009). Intrauterine growth retardation (IUGR): epidemiology and etiology. *Pediatric Endocrinology Reviews*.

[2] Gagnon R. (2003). Placental insufficiency and its consequences. *European Journal of Obstetrics & Gynecology and Reproductive Biology*.

[3] Roos S., Lagerlöf O., Wennergren M., Powell T. L., Jansson T. (2009). Regulation of amino acid transporters by glucose and growth factors in cultured primary human trophoblast cells is mediated by mTOR signaling. *American Journal of Physiology-Cell Physiology*.

[4] Gluckman P. D., Hanson M. A., Pinal C. (2005). The developmental origins of adult disease. *Maternal and Child Nutrition*.

[5] Simmons R. (2008). Perinatal programming of obesity. *Seminars in Perinatology*.

[6] Eriksson J. G. (2011). Early growth and coronary heart disease and type 2 diabetes: findings from the Helsinki Birth Cohort Study (HBCS). *The American Journal of Clinical Nutrition*.

[7] Salam R. A., Das J. K., Bhutta Z. A. (2014). Impact of intrauterine growth restriction on long-term health. *Current Opinion in Clinical Nutrition and Metabolic Care*.

[8] Liu X.-M., Kong J., Song W.-W., Lu Y. (2009). Glucose metabolic and gluconeogenic pathways disturbance in the intrauterine growth restricted adult male rats. *Chinese Medical Sciences Journal*.

[9] Schreuder M. F., Van Wijk J. A. E., Fodor M., Delemarre-van de Waal H. A. (2007). Influence of intrauterine growth restriction on renal function in the adult rat. *Journal of Physiology and Biochemistry*.

[10] Barnes S. K., Ozanne S. E. (2011). Pathways linking the early environment to long-term health and lifespan. *Progress in Biophysics and Molecular Biology*.

[11] Setia S., Sridhar M. G., Bhat V., Chaturvedula L., Vinayagamoorti R., John M. (2006). Insulin sensitivity and insulin secretion at birth in intrauterine growth retarded infants. *Pathology*.

[12] Brown L. D., Davis M., Wai S. (2016). Chronically increased amino acids improve insulin secretion, pancreatic vascularity, and islet size in growth-restricted fetal sheep. *Endocrinology*.

[13] Camacho L. E., Chen X., Hay W. W., Limesand S. W. (2017). Enhanced insulin secretion and insulin sensitivity in young lambs with placental insufficiency-induced intrauterine growth restriction. *American Journal of Physiology-Regulatory, Integrative and Comparative Physiology*.

[14] Ron D. (2002). Translational control in the endoplasmic reticulum stress response. *Journal of Clinical Investigation*.

[15] Xu C., Bailly-Maitre B., Reed J. C. (2005). Endoplasmic reticulum stress: cell life and death decisions. *Journal of Clinical Investigation*.

[16] Adachi Y., Yamamoto K., Okada T., Yoshida H., Harada A., Mori K. (2008). ATF6 is a transcription factor specializing in the regulation of quality control proteins in the endoplasmic reticulum. *Cell Structure and Function*.

[17] Lin J. H., Li H., Yasumura D. (2007). IRE1 signaling affects cell fate during the unfolded protein response. *Science*.

[18] Hirasawa H., Jiang C., Zhang P., Yang F.-C., Yokota H. (2010). Mechanical stimulation suppresses phosphorylation of eIF2 alpha and PERK-mediated responses to stress to the endoplasmic reticulum. *FEBS Letters*.

[19] Rasheva V. I., Domingos P. M. (2009). Cellular responses to endoplasmic reticulum stress and apoptosis. *Apoptosis*.

[20] Fonseca S. G., Burcin M., Gromada J., Urano F. (2009). Endoplasmic reticulum stress in beta-cells and development of diabetes. *Current Opinion in Pharmacology*.

[21] Soeda J., Mouralidarane A., Cordero P. (2016). Maternal obesity alters endoplasmic reticulum homeostasis in offspring pancreas. *Journal of Physiology and Biochemistry*.

[22] Soeda J., Cordero P., Li J. (2017). Hepatic rhythmicity of endoplasmic reticulum stress is disrupted in perinatal and adult mice models of high-fat diet-induced obesity. *International Journal of Food Sciences and Nutrition*.

[23] Riddle E. S., Campbell M. S., Lang B. Y. (2014). Intrauterine growth restriction increases TNF alpha and activates the unfolded protein response in male rat pups. *Journal of Obesity*.

[24] Liu X., Wang J., Gao L., Jiao Y., Liu C. (2018). Maternal protein restriction induces alterations in hepatic unfolded protein response-related molecules in adult rat offspring. *Front Endocrinol (Lausanne)*.

[25] Lee C. S., Sund N. J., Vatamaniuk M. Z., Matschinsky F. M., Stoffers D. A., Kaestner K. H. (2002). Foxa2 controls Pdx1 gene expression in pancreatic beta-cells in vivo. *Diabetes*.

[26] Deodati A., Argemi J., Germani D. (2018). The exposure to uteroplacental insufficiency is associated with activation of unfolded protein response in postnatal life. *PLoS One*.

[27] Liu X., Qi Y., Gao H. (2013). Maternal protein restriction induces alterations in insulin signaling and ATP sensitive potassium channel protein in hypothalami of intrauterine growth restriction fetal rats. *Journal of Clinical Biochemistry and Nutrition*.

[28] Wesolowski S. R., Hay W. W. (2016). Role of placental insufficiency and intrauterine growth restriction on the activation of fetal hepatic glucose production. *Molecular and Cellular Endocrinology*.

[29] Xing Y., Zhang J., Wei H. (2019). Reduction of the PI3K/Akt related signaling activities in skeletal muscle tissues involves insulin resistance in intrauterine growth restriction rats with catch-up growth. *PLoS One*.

[30] Hetz C. (2012). The unfolded protein response: controlling cell fate decisions under ER stress and beyond. *Nature Reviews Molecular Cell Biology*.

[31] Jaiswal H., Conz C., Otto H. (2011). The chaperone network connected to human ribosome-associated complex. *Molecular and Cellular Biology*.

[32] Ni M., Lee A. S. (2007). ER chaperones in mammalian development and human diseases. *FEBS Letters*.

[33] Schröder M., Kaufman R. J. (2005). ER stress and the unfolded protein response. *Mutation Research/Fundamental and Molecular Mechanisms of Mutagenesis*.

[34] Altmann S., Murani E., Schwerin M., Metges C. C., Wimmers K., Ponsuksili S. (2013). Dietary protein restriction and excess of pregnant German landrace sows induce changes in hepatic gene expression and promoter methylation of key metabolic genes in the offspring. *The Journal of Nutritional Biochemistry*.

[35] Zhang N. (2015). Epigenetic modulation of DNA methylation by nutrition and its mechanisms in animals. *Animal Nutrition*.

[36] Zhang K., Shen X., Wu J. (2006). Endoplasmic reticulum stress activates cleavage of CREBH to induce a systemic inflammatory response. *Cell*.

[37] Asada R., Kanemoto S., Kondo S., Saito A., Imaizumi K. (2011). The signalling from endoplasmic reticulum-resident bZIP transcription factors involved in diverse cellular physiology. *Journal of Biochemistry*.

[38] Vecchi C., Montosi G., Zhang K. (2009). ER stress controls iron metabolism through induction of hepcidin. *Science*.

[39] De Domenico I., Kaplan J. (2009). A new wrinkle in the fold: hepcidin links inflammation to the unfolded protein response. *Cell Metabolism*.

[40] Cnop M., Abdulkarim B., Bottu G. (2014). RNA sequencing identifies dysregulation of the human pancreatic islet transcriptome by the saturated fatty acid palmitate. *Diabetes*.

[41] B’chir W., Maurin A.-C., Carraro V. (2013). The eIF2*α*/ATF4 pathway is essential for stress-induced autophagy gene expression. *Nucleic Acids Research*.

[42] Sachdeva M. M., Claiborn K. C., Khoo C. (2009). Pdx1 (MODY4) regulates pancreatic beta cell susceptibility to ER stress. *Proceedings of the National Academy of Sciences*.

[43] Rashid C. S., Lien Y.-C., Bansal A. (2018). Transcriptomic analysis reveals novel mechanisms mediating islet dysfunction in the intrauterine growth-restricted rat. *Endocrinology*.

