# Supplementary material for: Dysregulation in the Unfolded Protein Response in the FGR Rat Pancreas
Source: Int J Endocrinol. 2020 Jan 20;2020:5759182. doi: 10.1155/2020/5759182 (PMC7201628; doi:10.1155/2020/5759182)
Supplement: Supplementary Materials — Supplement Table 1: composition of rat diets. Supplement Table 2: antibodies for western blotting, IHC, and IF. Supplement Table 3: primers for qPCR. Supplement Table 4: relative expression of the 84 UPR-related genes by PCR Arrays. [file 5759182.f1.pdf]

Supplement table 1. Composition of rat diets

	Standard diet (%)	LP diet (%)
Protein	22.2	7
Fat	4.8	6.9
Carbohydrates	61.2	74.7
Ca	1.62	0.53
P	0.92	0.3
Calories	399 kcal/100 g	395 kcal/100 g

LP, low protein

Supplement table 2. Antibodies for western blotting, IHC and IF

Target protein	Source	Company	Ref.	Dilution
Bip	Rabbit	CST	3177	1:1000
Atf6	Rabbit	Proteintech	24169-1	1:500
Hapa1l	Rabbit	Proteintech	13970-1	1:2000
Herpud1	Rabbit	Abcam	ab150424	1:1000
Creb3l3	Rabbit	Immunoway	YN1809	1:1000
Atf4	Rabbit	Proteintech	10835-1	1:1000
Perk	Rabbit	Immunoway	YT6126	1:1000
eIF2α	Rabbit	CST	5324	1:1000
p-eIF2α	Rabbit	CST	3398	1:500
Xbp1	Rabbit	Enogene	E90127	1:500
Jnk1	Rabbit	CST	3708	1:500
Pdx1	Rabbit	Enogene	E2A7170	1:500
Beclin1	Rabbit	Proteintech	11306-1	1:500
LC3	Rabbit	CST	2775	1:1000
Ampk	Rabbit	Enogene	E1A6423	1:1000
p-Ampk	Rabbit	Enogene	E1A3423	1:1000
Cleavage-caspase3	Rabbit	Absin	abs132005	1:1000
Bax	Rabbit	SAB	40635	1:1000
Bcl-2	Rabbit	Enogene	E1A6139	1:3000
Cleavage-caspase8	Rabbit	Enogene	E1A5267	1:2000
Cleavage-caspase9	Rabbit	Wanlei	WL01838	1:1000
Caspase-12	Rabbit	Wanlei	WL03268	1:500
Insulin	mouse	Proteintech	66198-1	1:100
β-actin	mouse	Proteintech	66009-1	1:5000

Supplement table 3. Primers for qPCR

Gene name	Accession Number	Primer Sequences (5'-3')	Size (bp)	Annealing temp (℃)
Atf2	NM_031018.1	GACAAACCATGCCCGTTGCT<br>GGACCGGTTGAGGAGAGGA	141	60
Atf4	NM_024403.2	CTTCTCCAGGTGTTCCTCGTT<br>GCTCAGCCCTCTTCTTCTG	162	60
Atf6	NM_001107196.1	GATTTGATGCCTTGGGAGTC<br>GGACCGAGGAGAAGAGACAG	122	60
Bip1	NM_013083.2	CCCAGGTCAAACACGAGGA<br>GCGGCAAGCAACCAAGGAT	137	60
Creb3l3	NM_001012115	ATAGCGGTCCTTCTGCTGTC<br>CTGTCGACTTTGTTGGCAGT	77	60
Chop	NM_001109986.1	GGCTTTGGGAGGTGCTTGTG<br>CTGCCTTTCGCCTTTGAGAC	201	60
Dnajc1	XM_002728514.4	AGACAGTGACCTCAGAAGCC<br>AGCTTCTCCTCTGGTTCCAC	88	60
Dnajc2	NM_053776	AGAGAAGTCAACCGGCAGAA<br>CCCATCTGGAGTTTGTCCCA	113	60
Grp94	NM_001012197.2	AAGGTCATTGTCACGTCGAAA<br>GTGTTTCCTCTTGGGTCAGC	97	60
Hapa1l	NM_212546.4	TCTCGTCCATGGTGCTGACC<br>GTGGCCTGTCTCTGGGAGTC	108	60
Herpud1	NM_053523	CGGACAACACTAGTCAGGCA<br>GCTTCAGGCCTAGAGACGTT	118	60
Perk	NM_031599.2	GTGCTCCGCTTATTCCTTT<br>CCTGTCTTGGTTGGGTCTG	158	60
Xbp1	NM_001004210.2	GATGAATGCCCTGGTTACTG<br>AGATGTTCTGGGGAGGTGAC	152	60
β-actin	NM_031144	AGTCCCTCACCCTCCCAAAAG<br>AAGCAATGCTGTCACCTTCCC	95	60

Supplement table 4. Relative expression of the 84 UPR-related genes by PCR Arrays

Gene Symbol	AVG DeltaCt		2^(-DeltaCt)		Fold Difference（F/C）	T-TEST (p value)	Up- or Down-Regulation（F/C）
	FGR	Control	FGR	Control			
**Atf4**	0.644444	1.89	0.639739	0.269807	2.3711	0.03574	2.3711
**Atf6**	4.527778	5.9	0.043351	0.016746	2.5887	0.02258	2.5887
**Atxn3**	6.067778	5.836667	0.014908	0.017498	0.852	0.49245	-1.1737
**Bax**	4.257778	4.14	0.052273	0.05672	0.9216	0.73868	-1.0851
**Calr**	-0.355556	-0.17	1.279478	1.125058	1.1373	0.64846	1.1373
**Canx**	1.227778	1.43	0.426975	0.371131	1.1505	0.64141	1.1505
**Cct4**	0.644444	0.65	0.639739	0.63728	1.0039	0.92961	1.0039
**Cct7**	0.904444	0.91	0.534238	0.532185	1.0039	0.90484	1.0039
**Cebpb**	9.411111	9.85	0.001469	0.001084	1.3556	0.46276	1.3556
**Creb3**	4.057778	3.823333	0.060046	0.070642	0.85	0.63122	-1.1765
**Creb3l1**	5.217778	5.52	0.026872	0.021793	1.233	0.44966	1.233
**Creb3l2**	5.464444	5.64	0.022648	0.020054	1.1294	0.55933	1.1294
**Creb3l3**	11.384444	8.776667	0.000374	0.00228	0.1641	0.02855	-6.0956
**Creb3l4**	7.134444	7.49	0.007117	0.005563	1.2795	0.44939	1.2795
**Ddit3**	5.041111	5.33	0.030372	0.024861	1.2217	0.35244	1.2217
**Derl1**	3.854444	4.466667	0.069135	0.045227	1.5286	0.17998	1.5286
**Dnajb9**	4.347778	4.913333	0.049112	0.033185	1.48	0.38759	1.48
**Dnajc10**	5.084444	5	0.029473	0.03125	0.9431	0.86299	-1.0603
**Dnajc3**	0.867778	1.503333	0.54799	0.352737	1.5535	0.20002	1.5535
**Dnajc4**	6.611111	6.71	0.01023	0.009552	1.0709	0.61378	1.0709
**Tor1a**	3.564444	4.14	0.084527	0.05672	1.4903	0.2162	1.4903
**Edem1**	2.354444	2.906667	0.195543	0.133354	1.4663	0.09532	1.4663
**Edem2**	2.984444	3.78	0.126355	0.072796	1.7357	0.24519	1.7357
**Eif2a**	4.447778	4.283333	0.045823	0.051356	0.8923	0.37229	-1.1207
**Eif2ak2**	6.337778	5.716667	0.012363	0.019016	0.6502	0.07429	-1.5381
**Eif2ak3**	3.141111	4.806667	0.113353	0.035731	3.1724	0.0135	3.1724
**Eif2ak4**	4.274444	4.513333	0.051673	0.043788	1.1801	0.40182	1.1801
**Ern2**	12.21777	12.79	0.00021	0.000141	1.4868	0.9304	1.4868
**Ero1a**	3.691111	3.696667	0.077422	0.077125	1.0039	0.96113	1.0039
**Fbxo6**	4.671111	4.666667	0.039251	0.039373	0.9969	0.93576	-1.0031
**Ganab**	2.544444	3.706667	0.171414	0.076592	2.238	0.0538	2.238
**Herpud1**	1.717778	3.33	0.304017	0.099442	3.0572	0.03835	3.0572
**Herpud2**	4.637778	4.603333	0.040169	0.041139	0.9764	0.95874	-1.0242
**Hsp90b1**	-0.758889	-0.41	1.692187	1.328686	1.2736	0.38418	1.2736
**Hspa1l**	10.481111	12.346667	0.0007	0.000192	3.6441	0.01559	3.6441
**Hspa2**	8.094444	8.233333	0.003659	0.003323	1.1011	0.63983	1.1011
**Hspa4**	3.107778	3.016667	0.116002	0.123564	0.9388	0.68541	-1.0652
**Hspa4l**	7.964444	8.063333	0.004004	0.003738	1.0709	0.97953	1.0709
**Hspa5**	-0.225556	0.383333	1.169227	0.766664	1.5251	0.37605	1.5251
**Hspb9**	12.671111	11.74	0.000153	0.000292	0.5245	0.13703	-1.9067
**Hsph1**	6.351111	6.196667	0.01225	0.013634	0.8985	0.72709	-1.113
**Htra2**	2.587778	3.066667	0.166342	0.119355	1.3937	0.06507	1.3937
**Htra4**	10.211111	9.546667	0.000844	0.001337	0.6309	0.21535	-1.585
**Insig1**	3.041111	3.166667	0.121488	0.111362	1.0909	0.78615	1.0909
**Insig2**	6.087778	6.043333	0.014703	0.015163	0.9697	0.93743	-1.0313
**Manf**	0.931111	1.476667	0.524454	0.359318	1.4596	0.2923	1.4596
**Mapk10**	9.374444	8.983333	0.001507	0.001976	0.7625	0.51065	-1.3114
**Mapk8**	5.441111	5.566667	0.023018	0.021099	1.0909	0.61933	1.0909

**Mapk9**	4.057778	4.2	0.060046	0.054409	1.1036	0.58014	1.1036
**Mbtps1**	3.627778	4.153333	0.080897	0.056198	1.4395	0.3491	1.4395
**Mbtps2**	5.567778	5.546667	0.021083	0.021394	0.9855	0.89622	-1.0147
**Nploc4**	4.091111	3.976667	0.058675	0.063519	0.9237	0.8021	-1.0826
**Nucb1**	3.287778	3.703333	0.102395	0.076769	1.3338	0.33322	1.3338
**Os9**	2.847778	3.47	0.13891	0.090246	1.5392	0.30407	1.5392
**Pdia3**	0.311111	0.583333	0.806021	0.66742	1.2077	0.56273	1.2077
**Pfdn5**	2.267778	2.256667	0.207649	0.209255	0.9923	0.8695	-1.0077
**Pfdn6**	2.941111	2.96	0.130208	0.128514	1.0132	0.82495	1.0132
**Ppia**	-2.508889	-2.623333	5.691815	6.161721	0.9237	0.54464	-1.0826
**Ppib**	-1.635556	-1.29	3.107072	2.445281	1.2706	0.29974	1.2706
**Ppic**	2.891111	2.963333	0.1348	0.128218	1.0513	0.61458	1.0513
**Ppp1r15a**	11.084444	11.403333	0.000461	0.000369	1.2474	0.25049	1.2474
**Ppp1r15b**	3.174444	3.736667	0.110764	0.075016	1.4765	0.3364	1.4765
**Prkcsh**	2.821111	3.206667	0.141501	0.108317	1.3064	0.31514	1.3064
**Rnf139**	3.464444	3.833333	0.090594	0.070154	1.2914	0.15107	1.2914
**Rpn1**	-0.592222	-0.193333	1.507567	1.143402	1.3185	0.32289	1.3185
**Scap**	2.271111	3.29	0.20717	0.102238	2.0264	0.29193	2.0264
**Sec62**	1.241111	1.41	0.423047	0.376312	1.1242	0.43421	1.1242
**Sec63**	3.044444	3.253333	0.121208	0.104869	1.1558	0.43616	1.1558
**Sel1l**	1.474444	2.87	0.359872	0.136787	2.6309	0.08426	2.6309
**Vimp**	2.161111	2.316667	0.223584	0.200731	1.1139	0.50979	1.1139
**Serp1**	-1.282222	-0.873333	2.432133	1.831891	1.3277	0.45539	1.3277
**Sil1**	5.341111	6.206667	0.02467	0.01354	1.822	0.07869	1.822
**Srebf1**	10.341111	10.51	0.000771	0.000686	1.1242	0.73574	1.1242
**Srebf2**	8.384444	9.073333	0.002992	0.001856	1.612	0.18618	1.612
**Syvn1**	3.894444	4.336667	0.067244	0.049492	1.3587	0.37585	1.3587
**Tcp1**	1.751111	1.703333	0.297073	0.307076	0.9674	0.92308	-1.0337
**Ube2g2**	5.804444	6.196667	0.017893	0.013634	1.3124	0.37226	1.3124
**Ube2j2**	4.667778	4.74	0.039342	0.037421	1.0513	0.6841	1.0513
**Ubxn4**	2.964444	3.493333	0.128119	0.088798	1.4428	0.03773	1.4428
**Ufd1l**	4.097778	4.423333	0.058404	0.046606	1.2531	0.17838	1.2531
**Uggt1**	3.954444	4.133333	0.064505	0.056983	1.132	0.71615	1.132
**Usp14**	3.154444	3.34	0.11231	0.098755	1.1373	0.30157	1.1373
**Vcp**	1.221111	1.533333	0.428952	0.345478	1.2416	0.30008	1.2416
**Xbp1**	1.317778	2.84	0.401152	0.139661	2.8723	0.09153	2.8723
**Actb**	-3.032222	-2.926667	8.180688	7.603516	1.0759	0.64805	1.0759
**B2m**	0.361111	0.353333	0.778565	0.782773	0.9946	0.97205	-1.0054
**Hprt1**	2.671111	2.573333	0.157006	0.168016	0.9345	0.36109	-1.0701
**Ldha**	1.571111	1.636667	0.336549	0.321599	1.0465	0.60043	1.0465
**Rplp1**	-3.375556	-2.89	10.378712	7.412704	1.4001	0.27865	1.4001

CT: threshold cycle; F/C: FGR/control relative expression.
